# Characterization and functional evaluation of JS207, a novel bispecific antibody against human PD-1 and VEGFA

**DOI:** 10.3389/fimmu.2025.1612547

**Published:** 2025-06-18

**Authors:** Shihua Lin, Min Hong, Jing Zhang, Wenting Zhao, Ke Li, Chun Wu, Qiuyun Yang, Yi Xiao, Lanqing Huang, Jing Wang, Aijuan Jia, Xujia Wang, Sheng Yao

**Affiliations:** ^1^ The R&D Department of Topalliance Biosciences, Inc., Rockville, MD, United States; ^2^ R&D Department, Suzhou Union Biopharm Co., Ltd., Suzhou, Jiangsu, China

**Keywords:** PD-1/PD-L1, vascular endothelial growth factor A (VEGFA), tumor microenvironment (TME), bispecific antibody, JS207, internalization, thermal stability

## Abstract

**Introduction:**

Cancer immunotherapy has been revolutionized by targeting PD-1 to restore antitumor T-cell activity and blocking VEGF to attenuate immunosuppressive tumor angiogenesis. While combining PD-1 and VEGF inhibition has shown promise in enhancing antitumor responses, co-administration of two or more monoclonal antibodies face several challenges, including distinct pharmacokinetics, complex dosing, and toxicity. A bispecific antibody (BsAb) targeting both PD-1 and VEGF pathways could overcome these limitations by enabling simultaneous, localized blockades of PD-1 and VEGF signaling within the tumor microenvironment (TME) as both PD-1 and VEGF are usually co-expressed in the TME.

**Methods:**

Here, we describe the in vitro characterization, functional and preclinical evaluation of JS207, a novel BsAb targeting PD-1 and VEGFA with high antigen binding affinity. JS207 matched or surpassed the activity of benchmarks antibodies in several in vitro binding assessments, T cell activation, VEGF signaling inhibition, cytokines (IL-2 and IFN-γ) release.

**Results:**

JS207 showed significant anti-tumor efficacy in mouse MC38 colon cancer model and A375 melanoma tumor model. Investigation into the mechanism of action revealed that VEGFA could significantly promote JS207’s antigen binding activity, T cell activation potency, and internalization of cell surface PD-1. *In vivo* results demonstrated that JS207 was well-tolerated and presented remarkable anti-tumor efficacy. In addition, JS207 showed enhanced thermal stability as evidenced by retained potency under heat stress, a critical factor for CMC (Chemistry, manufacturing and control) manufacture, storage and drug shelf life.

**Conclusion:**

JS207 is a promising therapeutic candidate that may address unmet clinical needs in cancer immunotherapy.

## 1 Introduction

Cancer persists as a leading cause of global mortality, with conventional therapies often failing to control advanced or metastatic diseases. The immune checkpoint inhibitors (ICIs)-based cancer immunotherapies have achieved remarkable success across cancers (1). Among these, targeting programmed cell death protein 1 (PD-1) and its ligand (PD-L1), have shown remarkable efficacy in various cancers (2). Yet, intrinsic, and adaptive resistance limits the efficacy of ICIs, with only 20–40% of patients achieving long-term remission (3, 4). A key driver of this resistance is the immunosuppressive tumor microenvironment (TME), a dynamic ecosystem comprising cancer cells, stromal cells, immune infiltrates, endothelial cells and aberrant vasculature that collaboratively foster immune evasion, angiogenesis, and metastatic spread (5, 6).

Within the TME, vascular endothelial growth factor (VEGF) plays a dual role, promoting tumor angiogenesis while suppressing antitumor immunity, such as, inhibiting dendritic cell maturation, recruiting regulatory T cells, and impairing cytotoxic T-cell activity (7). Vascular endothelial growth factor A (VEGFA) is a key member of the VEGF family proteins that can be secreted by various types of cells, including endothelial cells and tumor cells (8). VEGFA promotes angiogenesis mainly by binding to VEGF receptor-2 (VEGFR2). The engagement of VEGFR2 by VEGFA causes dimerization and intracellular autophosphorylation of VEGFR2, thereby activating downstream signaling pathways (9). This signaling cascade drives endothelial cell proliferation, migration, and survival, establishing a chaotic vascular network that sustains tumor growth and metastasis (6, 10). In solid tumors, angiogenesis plays a key role in tumor uptake of nutrients and oxygen, followed by proliferation and metastasis (11). Therapeutic antibodies against VEGFA specifically block the binding of VEGFA to VEGFR2 and exert anti-tumor effects (10).

Despite the success of PD-1 inhibitors and anti-VEGF therapies, significant challenges persist in monotherapies resulting in limited efficacy due to the complex and adaptive nature of tumors (12). Resistance mechanisms, such as upregulation of alternative angiogenic pathways or immune evasion tactics, frequently diminish the long-term effectiveness of these treatments (13). Additionally, the heterogeneity of tumors and TME mean that a single therapeutic target may not be sufficient to achieve comprehensive tumor control (14). The need for combination therapies has become evident, yet the concurrent administration of multiple agents can lead to increased toxicity and adverse effects (AEs), complicating patient management and reducing quality of life (15, 16).

PD-(L)1 and VEGF have been shown to be co-expressed in the TME of various tumor types. The expression of PD-(L)1 and VEGF could be used as biomarkers for selecting patients who may benefit from PD-(L)1 and VEGF inhibitors (17, 18). The concentration of VEGF is significant higher in the TME than in plasma (17, 19). This elevated concentration is due to the increased secretion of VEGF by tumor cells, which promotes angiogenesis and supports tumor growth (20, 21). Numerous studies have demonstrated that blocking VEGFA/VEGFR signal pathway could induce tumor regression by not only inhibiting the proliferation of endothelial cells and the formation of new blood vessels in the TME but also improving the infiltration of cytotoxic lymphocytes into the TME, while PD-1/PD-L1 pathway blockade could activate the infiltrated cytotoxic lymphocytes by removing the immunosuppressive effect mediated by this pathway (22). Dual targeting-PD-1 and VEGFR2 significantly inhibited primary tumor growth and doubled survival in murine models of hepatocellular carcinoma (23). Combining anti-VEGFA and anti-PD-1/L1 agents has shown promise in clinical beneficials, as seen in clinical trials pairing bevacizumab with atezolizumab (24, 25). However, conventional combination therapies face challenges, including discordant pharmacokinetics, overlapping toxicities, and dosing complexities (26, 27). The Food and Drug Administration AEs reporting system database showed that the combination of PD-(L)1 inhibitors with bevacizumab provided a survival benefit but significantly increased the risk of various AEs, including fever, neutropenia, nephritis, and thrombocytopenic purpura, which were attributed to the combination therapy as an independent risk factor for these AEs (27). Bispecific antibodies (BsAb), engineered to simultaneously target two antigens, represent a promising solution to these challenges by simultaneously targeting two distinct pathways (28). This dual-targeting approach can enhance therapeutic efficacy while potentially reducing toxicity and the likelihood of resistance development (29). In the context of cancer therapy, particularly in solid tumors, BsAbs provide a multifaceted approach to immunotherapy by simultaneously targeting PD-(L)1 and other immune regulatory molecules, such as anti-CD47/PD-L1, anti-PD-1/CTLA-4, and anti-4-1BB/PD-L1. This strategy could enhance antitumor immunity, mitigate immune evasion, and overcome the limitations of monotherapy approaches (19). BsAb that combine immune checkpoint inhibition with anti-angiogenic effects hold particular promise (28). Combinations of anti-PD-(L)1 and VEGFA inhibition have been clinically validated and approved for the treatment of solid tumors (30). For example, AK112, also known as ivonescimab, is the first-in-class humanized IgG1 bispecific antibody that targets PD-1 and VEGFA by inhibiting PD-1-mediated immunosuppression and simultaneously blocking tumor angiogenesis in the TME (31). By concurrently blocking PD-1-mediated immune evasion and VEGFA-driven angiogenesis, AK112 has demonstrated potent anti-tumor efficacy in both preclinical and clinical settings (31–33). Thus, through dual targeting PD-1-expressing T cells and VEGF-rich vasculature, dual target approach via BsAb could exert a more comprehensive anti-tumor effect.

Here, we describe a novel BsAb, JS207, designed to overcome resistance mechanisms in cancer therapy. JS207 is a recombinant humanized anti-PD-1 and VEGFA bispecific antibody of IgG4 κ subtype constructed in a tetravalent IgG-VHH format combining full-length anti-PD-1 IgG with VEGFA-targeting Variable domain of Heavy chain-only (VHH) antibody. This study characterized the physiochemical and biological properties of JS207 and evaluated the therapeutic potential of this novel anti-PD-1/anti-VEGF BsAb through assessing antigen binding affinity, T cell activation, HUVEC proliferation inhibition, and *in vivo* anti-tumor efficacy. Through these studies, we seek to establish a foundation for the clinical translation of this promising therapeutic approach, contributing to more effective and durable cancer treatments. In addition to extensive characterization studies, we compared JS207 with AK112 to assess the similarities and differences of these two BsAbs using a variety of methods to delineate the structural–functional relationships and antitumor activities. Our results demonstrated that engaging PD-1 and VEGFA by JS207 can significantly enhance antigen binding and PD-1 internalization, T cell activation, and anti-tumor activities.

## 2 Materials and methods

### 2.1 Materials, cell lines, and animals

The list of materials, cell lines and animals used in this study is available in the **Supplementary Materials** in **Supplementary Data Sheet**.

### 2.2 Enzyme-linked immunosorbent assay

Binding to PD-1: To assess the binding of JS207 to PD-1 and related proteins, 0.3 μg/mL of target protein (PD-1, BTLA, PD-L1, PD-L2 and ICOS, all from human) was coated onto 96‐well plates. After blocking with 2%BSA, test samples were then added. Bound antibody was detected using an HRP-goat anti‐human IgG (Fc‐specific) antibody. Absorbance was measured at 450 nm/620 nm. To assess the species cross-reactivity, PD-1 proteins from rat, mouse, Cynomolgus monkey and human were used (**Supplementary Data Sheet**).

Binding to VEGFA: To evaluate the binding of JS207 to VEGFA and related proteins, 0.3 μg/mL of target protein (VEGFA, VEGFB, VEGFC, VEGFD, VEGFE and PLGF, all from human) was coated onto 96‐well plates. After blocking with 2% BSA, test samples were added. Detection was carried out using an HRP-goat anti‐human IgG (Fc‐specific) antibody. To assess the species cross-reactivity, VEGFA proteins from rat, mouse, human and Cynomolgus monkey were used (**Supplementary Data Sheet**).

Blocking the interaction between human PD-1 with PD-L1/PD-L2: Inhibition of the interaction between human PD-1 and its ligands (PD-L1 and PD-L2) was assessed using blocking ELISAs. Human PD-1 protein (1.5 μg/mL) was coated on the plate and blocked with 2% BSA. Test samples were prepared in assay buffer containing biotin‐PD-L1 hFc (4.0 μg/mL). After detection with HRP-streptavidin, absorbance was read at 450 nm/620 nm (**Supplementary Data Sheet**).

Blocking the interaction between human VEGFA and VEGFR2: To assess JS207’s competitive inhibition of the VEGFA–VEGFR2 interaction, 0.5 μg/mL of human VEGFA was coated on 96‐well plates and blocked with 2% BSA. Test samples (JS207, VEGF-DotAb, AK112) were added in assay buffer containing biotin‐VEGFR2 (0.3 μg/mL). After detection with HRP-streptavidin, absorbance was read at 450 nm/620 nm (**Supplementary Data Sheet**).

### 2.3 Surface plasma resonance binding assays

Binding to human PD-1: The binding affinity of JS207 to human PD-1 was measured by Biacore T200. 40 µg/mL of anti-human Fc antibody was coated to CM5 chip, and then 2 μg/mL of JS207 were captured. Serially diluted human PD-1 was then applied, and the kinetic model was analyzed, and the binding affinity (K_D_ value) was calculated (**Supplementary Data Sheet**).

Binding to human VEGFA: The binding affinity of JS207 to human VEGFA was determined by the Biacore T200. 40 µg/mL of anti-human Fc antibody was coated to CM5 chip, and test samples were added. The kinetic model was analyzed, and the binding affinity (K_D_ value) was calculated (**Supplementary Data Sheet**).

Binding of JS207/VEGFA complex to human PD-1: The binding of pre-formed JS207/VEGFA complex to human PD-1 was assessed using the Octet RED96e system. 20 µg/mL of human PD-1-mFc was immobilized on an AMC biosensor, then incubated with JS207/VEGFA complex. Kinetic parameters were analyzed using Data Analysis 11.1 software (**Supplementary Data Sheet**).

Simultaneous binding of JS207 to human PD-1 and VEGFA: The ability of JS207 to simultaneously bind to human PD-1 and human VEGFA was measured using the Biacore T200. Two different experimental formats were used: (1) PD-1 first, then VEGFA, and (2) VEGFA first, then PD-1 (**Supplementary Data Sheet**).

### 2.4 Luciferase reporter gene assays

PD-1 reporter gene assay: Jurkat/PD-1-NAFT-Luc (Jurkat/PD-1) cells and PD-L1 aAPC/CHO-K1 (CHO/PD-L1) cells were used in this assay. JS207 inhibits the binding of PD-1 in Jurkat/PD-1 cells to PD-L1 in CHO/PD-L1 cells leading to NFAT/luciferase reporter gene activation, and the anti-PD-1 potency was determined via bioluminescent measurement (**Supplementary Data Sheet**). The signal to noise (S/N) ratio was generated by dividing the Top response by Bottom response. EC_50_ or IC_50_ is the concentration of an antibody required to achieve 50% of its maximum biological effect.

VEGF reporter gene assay: H293/VEGFR2 cell that was engineered to express VEGFR2/NFAT-luciferase was used in this assay. When VEGFA binds to VEGFR2 to initiate the signaling pathway, the NFAT-luciferase reporter gene is activated. The anti-VEGFA potency of JS207 was determined via bioluminescent measurement (**Supplementary Data Sheet**).

### 2.5 HUVEC proliferation inhibition assay

VEGFA at 10 ng/mL and the test antibody solutions at 0.004 nM-10 nM were added to 96-well plate and incubated at 37°C for 30 minutes. Then, HUVECs at 3×10^3^ cells/well were seeded and incubated at 37°C for 96 hours. Cell counting-Lite luciferase assay reagent was added and chemiluminescence signals were measured (**Supplementary Data Sheet**).

### 2.6 Flow cytometry binding experiments

Internalization assay using cell surface residual PD-1 quantification method: H293/PD-1 cells were seeded at 1×10^5^ cells/well in a 96-well plate. Serially diluted test samples were added in the absence or presence of VEGFA. After incubation at 4°C for 30-minutes, the cells were divided into two aliquots and incubated at 37°C and 4°C for 0.5, 1, 2, and 4 hours, respectively. All cells were stained with an anti-human IgG-PE antibody for 30 minutes at 4°C. The samples were then analyzed by flow cytometry. The mean fluorescence intensity (MFI) was determined, and the internalization index was calculated using the following formula (**Supplementary Data Sheet**):


Internalization Index = [1 − (MFI at 37°C)/(MFI at 4°C)] x 100.

Internalization assay using intracellular fluorescence method: JS207 and other test antibodies were conjugated with CypHer5E follow manufacture’s instruction (GE Healthecare). The conjugated antibodies were serially diluted and incubated with or without VEGFA, then incubated with Jurkat/PD-1 cells for 4 hours at either 4°C or 37°C. Cells were washed with cold medium and subsequently stained with a PE-labeled noncompetitive anti-PD-1 antibody (MIH4 PD-1 PE, BD Biosciences). Samples were then analyzed on a BD FACSCanto II flow cytometer (**Supplementary Data Sheet**).

Cell-based PD-1 binding: To assess the effect of VEGFA on cell-based PD-1 binding, H293/PD-1 cells were seeded at 1×10^5^ cells/well in a 96-well plate. Test samples were added in the absence or presence of VEGFA. The cells were incubated at 4°C for 30 minutes, stained with mouse anti-human Fc-PE antibody for 30 minutes at 4°C and then analyzed by flow cytometry. Data analysis was performed using FlowJo (**Supplementary Data Sheet**).

### 2.7 Mixed lymphocyte reaction assays

The effect of JS207 on IL-2 and IFN-γ release was evaluated using a mixed lymphocyte reaction (MLR) system. Peripheral blood mononuclear cells (PBMCs) resuspended in EasySep buffer and CD4^+^ T cells were isolated. Mature dendritic cells (mDCs) and purified CD4^+^ T cells were then seeded into 96-well plates at densities of 10,000 mDCs/well and 100,000 CD4^+^ T cells/well, respectively. Test samples were added with a final concentration ranging from 150 nM to 15 pM. The cells were incubated at 37 °C for 5 days. Supernatants were collected on days 3 and 5 for IL-2 and IFN-γ measurement (**Supplementary Data Sheet**).

### 2.8 Thermal stability assessment

Test samples were first diluted to 2 mg/mL in cell culture medium and then subjected to heat stress at 40°C for 0–96 hr, 55°C for 0 – 48hr, and 65°C for 0–4 hr to assess the impact of heat-stress on anti-PD-1 activity. To assess the impact of heat-stress on anti-VEGFA activity, samples were stressed at 40°C and 50°C for 0–6 days. The potency values of stressed samples was compared with the respective control samples (Time 0).

### 2.9 Anti-tumor activity studies

Mouse colon cancer MC38 model in B-hPD-1 humanized mice: MC38 cells were implanted subcutaneously in the right flank of C57BL/6-Pdcd1^tm1(PDCD1)Bcgen/Bcgen^ mice (B-hPD-1 humanized mice) at 1×10^5^ cells/mouse. When the mean tumor volume reached approximately 115 mm³, mice were randomly assigned into seven groups (n = 8 per group): (1) saline, (2) toripalimab 0.6 mg/kg, (3) VEGF-DotAb 0.33 mg/kg, (4) toripalimab 0.6 mg/kg + VEGF-DotAb + 0.33 mg/kg, (5) JS207 0.75 mg/kg, (6) JS207 1.5 mg/kg, and (7) JS207 4.5 mg/kg. Treatments were administered intraperitoneally twice weekly for a total of 6 doses. Tumor volumes and animal body weights were recorded (**Supplementary Data Sheet**).

Malignant melanoma A375 model in NDG mice: A375 cells at 5×10^6^ cells/mouse were suspended mixed with Matrigel and implanted subcutaneously into NDG mice. When the average tumor volume reached approximately 137 mm³, 10×10^6^ PBMCs in 0.2 mL were injected intravenously. Two days after PBMC administration, the tumor-bearing mice were randomly assigned to five groups (n = 8 per group): (1) saline control group, (2) AK112 11.1 mg/kg, (3) JS207 1.0 mg/kg), (4) JS207 3.0 mg/kg, and (5) JS207 10.0 mg/kg. All the treatments were administered intraperitoneally twice a week for a total of 6 doses (**Supplementary Data Sheet**). Tumor volumes and animal body weights were recorded, and tumor growth inhibition rate (TGI_TV_) was calculated as:


$$\text{TG}{\text{I}}_{\text{TV}}\left(\%\right)\;=\;\left[1-\left(\text{Ti}-\text{T}0\right)/\left(\text{Vi}-\text{V}0\right)\right]\times 100\%$$

Where: Ti = mean tumor size of the treatment group on the i-th day of administration, T0 = mean tumor size of the treatment group on day 0 of administration; Vi = mean tumor size of the negative control group on the i-th day of administration, V0 = mean tumor size of the negative control group on day 0 of administration.

All animals of the above *in vivo* studies were housed in an SPF-grade facility of Suzhou Junmeng Biopharmaceuticals. The housing conditions were maintained at a temperature of 20–26 °C, relative humidity of 40%-70%, with 12-hour light/dark cycle. All protocols and procedures are approved by the local Institutional Animal Care and Use Committee (IACUC) under permit number YTSYDWIACUC202401.

### 2.10 Statistical analysis

Statistical significance was determined using Microsoft Excel and GraphPad Prism software. Comparisons between groups were performed using a two-tailed Student’s t-test. Data are presented as the mean ± standard deviation, and differences between groups were considered statistically significant when p < 0.05 (*p < 0.05; **p < 0.01, ***p < 0.001, ****p < 0.0001).

## 3 Results

### 3.1 Structure and antigen binding profile of JS207

**Figure 1:**
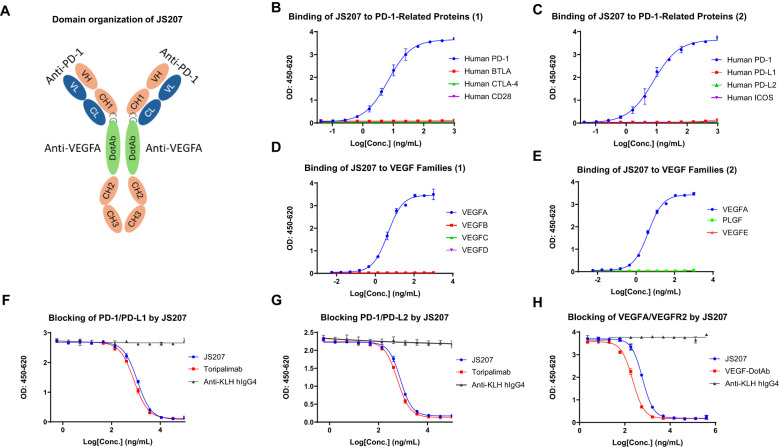
Structure and binding profile of JS207. **(A)** Domain organization of JS207. **(B)** JS207 binds to human PD−1 but not to BTLA, CTLA−4, or CD28. **(C)** JS207 binds to human PD−1 but not to PD−L1, PD−L2, or ICOS. **(D)** JS207 binds to human VEGFA but not to VEGFB, VEGFC, or VEGFD. **(E)** No binding of JS207 to human PLGF (placental growth factor) or VEGFE. **(F)** Blocking of PD−1/PD−L1 interaction by JS207 and toripalimab (positive control). **(G)** Blocking of PD−1/PD−L2 interaction by JS207. **(H)** Blocking of VEGFA/VEGFR2 interaction by JS207 and VEGF−DotAb (positive control). An anti−KLH hIgG4 antibody served as the negative control in experiments **(F–H)**.

JS207 is a recombinant humanized bispecific antibody that targets both PD−1 and VEGFA and belongs to the IgG4 κ subtype. It was independently developed by Shanghai Junshi Biosciences Co., Ltd. for the treatment of advanced malignancies. JS207 comprises two identical light chains (LC) and two identical heavy chains (HC), which are linked by intra− and inter−chain disulfide bonds. The molecule incorporates the Fab, hinge, and Fc regions derived from an anti−PD−1 monoclonal antibody, with an anti−VEGFA nanobody fused to the hinge region of the heavy chain via flexible linkers ((G_4_S)_3_ and (G_4_S)_1_) (**Figure 1A**). The intact molecular weight of JS207 is approximately 180.5 kDa.

### 3.2 Binding of JS207 to human PD-1 related immune proteins

The binding activity of JS207 to human PD−1 and a panel of related immune proteins was examined by ELISA. As shown in **Figures 1B, C**, JS207 exhibited concentration−dependent binding to human PD−1. In contrast, it did not bind to human BTLA, CTLA−4, CD28, PD−L1, PD−L2, or ICOS. Comparative analysis using toripalimab (an in−house, commercially approved anti−PD−1 antibody, also known as JS001) and AK112 demonstrated that all three antibodies specifically bound to human PD−1. The calculated EC_50_ values were 10.4 ng/mL (58.6 pM) for JS207, 8.9 ng/mL (60.5 pM) for toripalimab, and 20.1 ng/mL (100.0 pM) for AK112.

### 3.3 Binding of JS207 to human VEGF family proteins

The binding of JS207 to human vascular endothelial growth factor (VEGF) family proteins, including VEGFA, VEGFB, VEGFC, VEGFD, VEGFE, and placental growth factor (PLGF), was evaluated by ELISA. As shown in **Figures 1D, E**, JS207 specifically bound to human VEGFA, with no detectable binding to human VEGFB, VEGFC, VEGFD, VEGFE, or PLGF.

### 3.4 Blocking of PD-1/PD-L1, PD-1/PD-L2 and VEGFA/VEGFR2 by JS207

The ability of JS207 to inhibit the binding of human PD-1 to its ligands (PD-L1 and PD-L2) and to block the interaction between human VEGFA and VEGFR2 was assessed using blocking ELISA. In these assays, toripalimab and VEGF-DotAb (an in-house anti-VEGFA Dotbody) served as positive controls, while an anti-KLH hIgG4 antibody was used as the negative control.

As presented in **Figures 1F, G**, JS207 effectively blocked the interaction between human PD-1 and PD-L1 with an IC_50_ of 1149 ng/mL (6.37 nM) and between PD-1 and PD-L2 with an IC_50_ of 776.6 ng/mL (4.3 nM). Moreover, JS207 inhibited the binding of human VEGFA to VEGFR2 with an IC_50_ of 603.9 ng/mL (3.35 nM). In contrast, toripalimab only blocked the PD-1/PD-L1 and PD-1/PD-L2 interactions, with IC_50_ values of 883.7 ng/mL (5.56 nM) and 587.7 ng/mL (3.92 nM), respectively, but did not block the VEGFA/VEGFR2 interaction. VEGF-DotAb specifically inhibited VEGFA binding to VEGFR2, with an IC_50_ of 221.6 ng/mL (1.48 nM) (**Figure 1H**).

### 3.5 Species cross-reactivity of JS207

**Figure 2:**
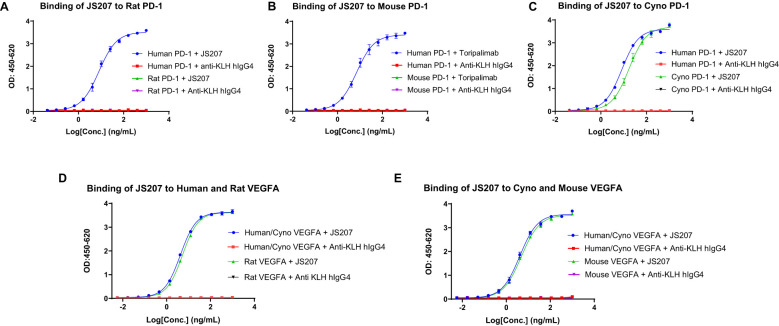
Species cross-reactivity of JS207. **(A)** No binding of JS207 to rat PD-1. **(B)** No binding of JS207 to mouse PD-1. **(C)** JS207 binding to human and Cynomolgus Monkey (Cyno) PD-1. **(D)** JS207 binding to human and rat VEGFA. **(E)** JS207 binding to Cyno and mouse VEGFA. Anti KLH hIgG4 was used as negative control.

To evaluate the cross-species reactivity, the binding of JS207 to PD-1 proteins from different species was assessed by ELISA. As shown in **Figures 2A–C**, JS207 bound potently to human PD-1 and cynomolgus (cyno) PD-1 with EC_50_ values of 8.2 ng/mL (45 pM) and 17.2 ng/mL (95 pM), respectively, while no binding was observed for rat or mouse PD-1. The negative control anti-KLH IgG4 exhibited no binding.

Similarly, the cross-species binding of JS207 to VEGFA was examined. As depicted in **Figures 2D, E**, JS207 bound to human and cyno VEGFA with an EC_50_ of 4.2 ng/mL (23 pM) and also recognized rat and mouse VEGFA with EC_50_ values of 5.3 ng/mL (29 pM) and 4.8 ng/mL (27 pM), respectively.

### 3.6 Binding affinity to human PD-1 and VEGFA

**Figure 3:**
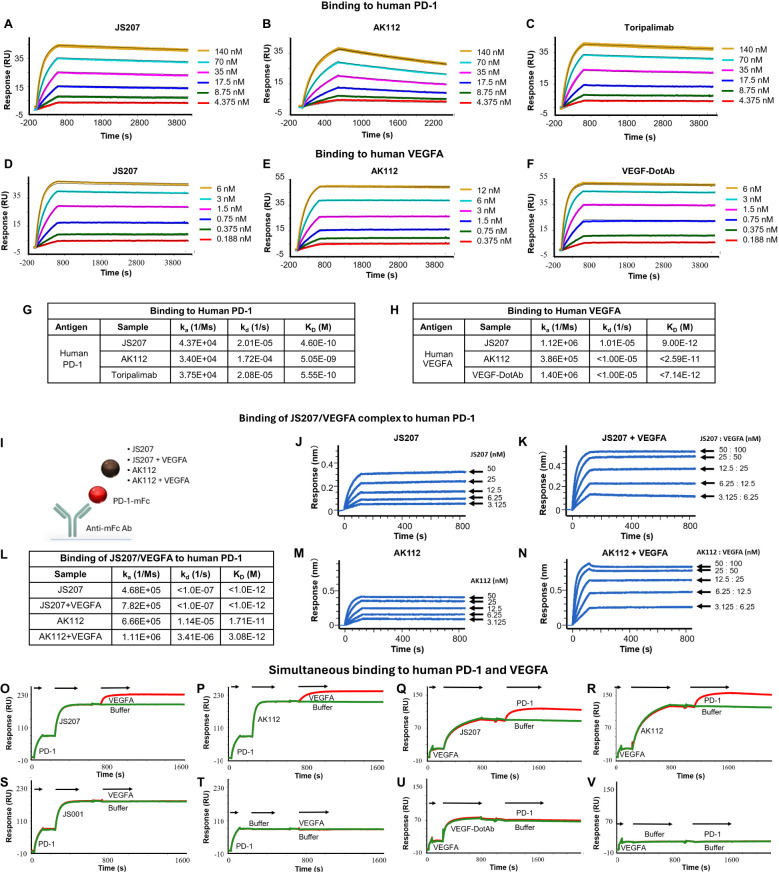
Antigen binding characteristics of JS207 in comparison with AK112, toripalimab and VEGF-DotAb. **(A)** JS207 binding to human PD-1. **(B)** AK112 binding to human PD-1. **(C)** Toripalimab binding to human PD-1. **(D)** JS207 binding to human VEGFA. **(E)** AK112 binding to human VEGFA. **(F)** VEGF-DotAb binding to human VEGFA. **(G)** Binding affinity of JS207, AK112 and toripalimab to human PD-1. **(H)** Binding affinity of JS207, AK112 and VEGF-DotAb to human VEGFA. **(I)** Experimental design for JS207/VEGFA and AK112/VEGFA complex binding to human PD-1. **(J)** JS207 binding to human PD-1. **(K)** JS207/VEGFA complex binding to human PD-1. **(L)** Binding affinity of JS207/VEGFA and AK112/VEGFA complex to human PD-1. **(M)** AK112 binding to human PD-1. **(N)** AK112/VEGFA complex binding to human PD-1. **(O)** JS207 binding to human PD-1, then human VEGFA. **(P)** AK112 binding to human PD-1, then human VEGFA. **(Q)** JS207 binding to human VEGFA, then human PD-1. **(R)** AK112 binding to human VEGFA, then human PD-1. **(S)** Toripalimab binding to human PD-1 but not VEGFA. **(T)** No binding in buffer control. **(U)** VEGF-DotAb binding to human VEGFA but not PD-1. **(V)** No binding signal for buffer control.

The binding affinity and kinetics of JS207 toward human PD-1 were characterized using surface plasmon resonance (SPR). **Figure 3A** shows the binding profile of JS207 to human PD-1, which is similar to that of toripalimab (**Figure 3C**), a finding that is consistent with the fact that the anti-PD-1 domain of JS207 is derived from toripalimab. In contrast, AK112 displayed a distinct profile (**Figure 3B**), with slower association and faster dissociation kinetics compared to JS207 and toripalimab. The equilibrium dissociation constant (K_D_) for JS207 binding to human PD-1 was determined to be 4.60×10^-10^ M, approximately 11-fold higher affinity than that of AK112 (5.05×10^-9^ M). In addition, the PD-1 binding affinity of JS207 was comparable to that of toripalimab (5.55×10^-10^ M) (**Figure 3G**).

The binding profile of JS207 to human VEGFA was similarly assessed by SPR. As shown in **Figures 3D–F**, the binding traces of JS207 were similar to those observed for AK112 and VEGF-DotAb. All three antibodies exhibited very high affinities for human VEGFA, with K_D_ values of 9.00×10^-12^ M for JS207, <2.59×10^-11^ M for AK112, and <7.14×10^-12^ M for VEGF-DotAb (**Figure 3H**).

### 3.7 Binding of JS207/VEGFA complex to human PD-1

Because JS207 exhibits high affinity for human VEGFA, intravenous administration is expected to result in the rapid formation of a JS207/VEGFA complex in circulation prior to its engagement with PD-1 within the tumor microenvironment. To determine whether this preformed complex retains its ability to bind human PD-1, we employed an Octet-based assay (**Figure 3I**). In this experiment, human PD-1 conjugated with a mouse Fc fragment was immobilized on an AMC biosensor. Next, JS207, the preformed JS207/VEGFA complex, AK112, and the AK112/VEGFA complex were injected (**Figures 3I–N**). As shown in **Figure 3K**, the JS207/VEGFA complex produced a strong binding signal to human PD-1, with a K_D_ value of <1.0×10^-12^ M, comparable to JS207 alone (**Figures 3J–L**). Similarly, the AK112/VEGFA complex bound to PD-1 with an affinity similar to that of AK112 alone (**Figures 3L–N**). These results indicate that both JS207/VEGFA and AK112/VEGFA complexes maintain robust binding activity to human PD-1.

### 3.8 Simultaneous binding of JS207 to human PD-1 and human VEGFA

To assess whether JS207 can bind human PD-1 and human VEGFA simultaneously, we designed an SPR assay. In this experiment, AK112 was used as a comparator, while toripalimab and VEGF-DotAb served as positive controls for PD-1 and VEGFA binding, respectively. In one format, human PD-1 was first captured; subsequent injection of JS207, followed by VEGFA, produced an additional binding response (**Figure 3O**), demonstrating that JS207 can engage both antigens simultaneously. A similar dual-binding profile was observed for AK112 (**Figure 3P**). In contrast, toripalimab bound exclusively to PD-1 (**Figure 3S**), and no binding was detected in the buffer control (**Figure 3T**). In a complementary approach, when human VEGFA was immobilized first, JS207 was subsequently able to bind human PD-1, further confirming JS207’s dual-binding capability (**Figures 3Q, R**). AK112 exhibited a similar binding pattern as JS207 (**Figures 3P, R**). As expected, toripalimab bound only to PD-1, while VEGF-DotAb bound exclusively to VEGFA (**Figure 3U**); no binding signal was observed in the corresponding buffer control (**Figure 3V**).

### 3.9 Anti-PD-1 potency of JS207 using PD-1 reporter gene assay

**Figure 4:**
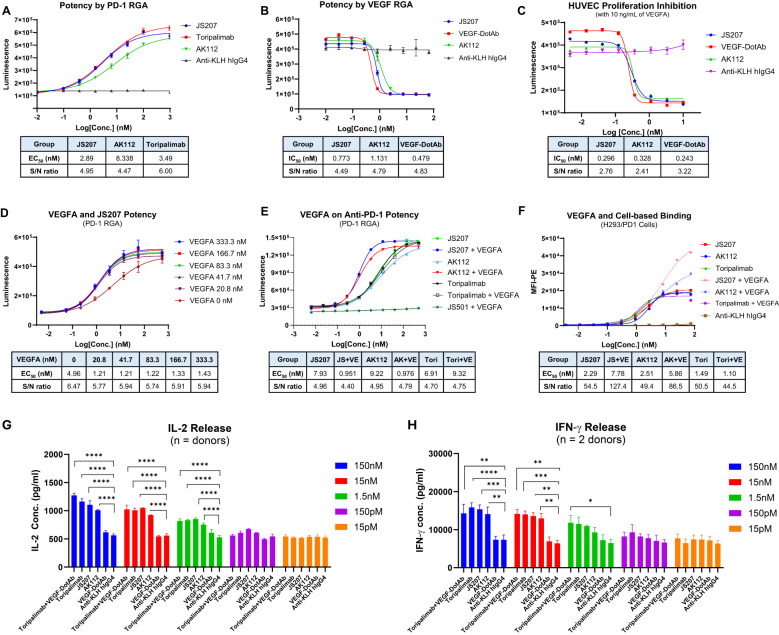
Cell-based biological activity of JS207. **(A)** Anti-PD-1 potency of JS207 in PD-1 reporter gene assay (RGA). **(B)** Anti-VEGFA potency of JS207 in VEGFA RGA. **(C)** HUVEC proliferation inhibition by JS207. **(D)** The impact of VEGFA concentration on JS207’s anti-PD-1 potency. **(E)** VEGFA enhanced anti-PD-1 activity of JS207 and AK112 but not toripalimab. **(F)** VEGFA enhanced cell-based PD-1 binding by JS207 and AK112. **(G)** JS207, AK112, toripalimab, and toripalimab plus VEGF-DotAb at 1.5 nM or higher significantly enhanced IL-2 levels. **(H)** JS207, AK112 and toripalimab at 15 nM or higher significantly enhanced IFN-γ levels. Toripalimab plus VEGF-DotAb at 1.5 nM significantly enhanced IFN-γ release in the MLR system. *p < 0.05; **p < 0.01, ***p < 0.001, ****p < 0.0001 vs negative control (anti-KLH IgG4) group.

The anti−PD−1 potency of JS207 was evaluated using a PD−1/PD−L1 reporter gene assay (RGA). Jurkat effector cells stably overexpressing human PD−1 and an NFAT−driven luciferase reporter were co−cultured with CHO target cells expressing human PD−L1. JS207 effectively blocked the PD−1/PD−L1 interaction, thereby promoting T cell activation. The EC_50_ value for JS207 was 2.89 nM with a signal-to-noise (S/N) ratio of 4.95. In parallel, the EC_50_ values and S/N ratios for AK112 and toripalimab were 8.34 nM, 4.47 and 3.49 nM, 6.0, respectively. Hence, the anti−PD−1 potency of JS207 is comparable to that of toripalimab and superior to that of AK112 (**Figure 4A**).

### 3.10 Anti-VEGFA potency of JS207 in VEGFA reporter gene assay

The anti−VEGFA activity of JS207 was assessed using a VEGFA/VEGFR2 RGA in H293 cells engineered to overexpress VEGFR2. As shown in **Figure 4B**, JS207, AK112, and VEGF−DotAb effectively inhibited the binding of VEGF to VEGFR2. The IC_50_ value for JS207 was 0.773 nM with an S/N ratio of 4.49; for AK112, the corresponding values were 1.131 nM and 4.79; and for VEGF−DotAb, 0.479 nM and 4.83, respectively. Thus, JS207 exhibited anti−VEGF activity comparable to or slightly better than that of AK112 but was marginally less potent than VEGF−DotAb (**Figure 4B**).

### 3.11 HUVEC proliferation inhibition by JS207

The ability of JS207 to inhibit human umbilical vein endothelial cell (HUVEC) proliferation was examined *in vitro*. HUVECs were cultured in the presence of 10 ng/mL VEGFA along with serial dilutions of JS207, AK112, and VEGF−DotAb (0.005–10.0 nM). The IC_50_ values and S/N ratios were determined to be 0.296 nM, 2.76 for JS207, 0.328 nM, 2.41 for AK112, and 0.243 nM, 3.22 for VEGF−DotAb. These data indicate that the inhibitory effect of JS207 on HUVEC proliferation is comparable to or marginally better than that of AK112, albeit somewhat less potent than VEGF−DotAb (**Figure 4C**).

### 3.12 VEGFA enhances JS207’s cell-based binding and anti-PD-1 potency

The effect of VEGFA on the anti−PD−1 activity of JS207 was investigated using the PD−1 RGA. As indicated in **Figure 4D**, the presence of 20.8 nM VEGFA markedly enhanced the anti−PD−1 activity of JS207 compared with JS207 alone. Further increasing the VEGFA concentration to 333.3 nM elevated the maximum response (upper asymptote) of JS207’s anti−PD−1 potency. VEGFA at 1000 nM also enhanced the anti−PD−1 potency of AK112 with similar extent as JS207, whereas toripalimab remained unaffected (**Figure 4E**).

Cell−based binding studies using H293/PD−1 cells demonstrated that VEGFA enhanced the binding of both JS207 and AK112 to PD−1, while toripalimab showed no such effect (**Figure 4F**). Similar results were observed in PD-1 expressing Jurkat/PD−1 cells (**Supplementary Figure S1** in **Supplementary Data Sheet**).

### 3.13 JS207 induces IL-2 and IFN-γ in the mixed lymphocytes reaction

The mixed lymphocyte reaction (MLR) system was used to assess JS207’s effect on T cell activation. In this assay, *in vitro*–induced mature dendritic cells and CD4^+^ T cells from four donors were co−incubated with JS207 and control antibodies over a concentration range of 15 pM to 150 nM. JS207 significantly promoted the release of IL−2 and IFN−γ in a dose−dependent manner. The cytokine responses induced by JS207 were comparable to those observed for AK112, toripalimab, and the combination of toripalimab plus VEGF−DotAb (**Figures 4G, H**). Notably, VEGF−DotAb alone did not induce IL−2 or IFN−γ release relative to the anti−KLH hIgG4 control.

### 3.14 JS207 induces PD-1 internalization

To investigate PD−1 internalization, two complementary methods were employed: the cell surface residual PD−1 assay and the intracellular fluorescence assay.

**Figure 5:**
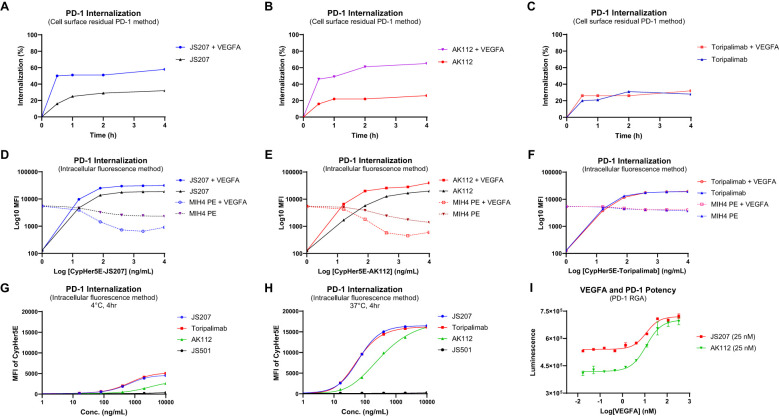
PD-1 internalization assessment using the cell surface residual PD-1 method in PD-1 expressing H293 cells **(A-C)** and the intracellular fluorescence method in Jurkat/PD-1 cells **(D-H)**. **(A)** VEGFA enhanced PD-1 internalization induced by JS207. **(B)** VEGFA enhanced PD-1 internalization induced by AK112. **(C)** VEGFA did not impact PD-1 internalization induced by toripalimab. **(D)** VEGFA enhanced JS207-induced PD-1 internalization with increased intracellular fluorescence and decreased cell surface PD-1 signal. **(E)** VEGFA enhanced AK112-induced PD-1 internalization with increased intracellular fluorescence and decreased cell surface PD-1 signal. **(F)** VEGFA did not impact PD-1 internalization induced by toripalimab. **(G)** Minimal PD-1 internalization was seen in cells incubated at 4°C. **(H)** JS207 and toripalimab showed comparable PD-1 internalizations, which are stronger than AK112. **(I)** VEGFA concentration-dependently enhanced anti-PD-1 potency of JS207 and AK112. The concentration of JS207 and AK112 used in this study was 25 nM.

Cell Surface Residual PD-1 Method: Human PD−1–expressing H293 cells were incubated at 4°C with 30 nM of JS207, AK112, or toripalimab for 30 minutes in the presence or absence of VEGFA (60 nM). After washing to remove unbound antibodies, cells were stained with PE−labeled anti−PD−1 human IgG to quantify the residual cell surface–bound PD−1. In the absence of VEGFA, approximately 16–20% of surface PD−1 was internalized within 30 minutes, increasing to 21–32% after 60–240 minutes of incubation for all three antibodies. However, in the presence of VEGFA, JS207− and AK112−treated cells exhibited a marked increase in PD-1 internalization, reaching 46–50% within 30 minutes and up to 65% after 4 hours, while toripalimab was unaffected (**Figures 5A–C**).

Intracellular Fluorescence Method: JS207, AK112, toripalimab, and JS501 (an anti−VEGFA mAb) were conjugated with CypHer5E, a pH−sensitive cyanine dye that exhibits enhanced fluorescence in acidic intracellular compartments. Jurkat/PD−1 cells were incubated with serial dilutions of these CypHer5E−conjugated antibodies at 37°C for 4 hours. At the end of incubation, a noncompetitive anti−PD−1 antibody (MIH4 PD−1−PE) was added to measure the remaining cell surface PD−1. As shown in **Figures 5D–F**, the intracellular fluorescence intensity of CypHer5E−labeled JS207 and AK112 increased in a dose−dependent manner, while the cell surface PD−1 signal detected by MIH4 PD−1−PE concomitantly decreased, providing further evidence of PD−1 internalization. In the presence of VEGFA, both fluorescence intensity and PD−1 internalization were further enhanced for JS207 and AK112. In contrast, toripalimab–induced internalization was not affected by VEGFA (**Figure 5F**). Only minimal internalization was observed when cells were incubated at 4°C (**Figure 5G**).

In the absence of VEGFA, JS207 and toripalimab exhibited comparable internalization activities with EC_50_ values of 62.3 ng/mL (0.35 nM) and 58.4 ng/mL (0.39 nM), respectively, both of which were more potent than AK112 (EC_50_ = 322.7 ng/mL, or 1.61 nM). To assess the impact of varying VEGFA concentrations on PD−1 internalization, a PD−1 RGA was performed using a fixed concentration of JS207 (25 nM) and AK112 (25 nM). As shown in **Figure 5I**, when VEGFA concentrations were low (0.017–1.37 nM), JS207 exhibited higher anti-PD−1 potency than AK112. However, at high VEGFA concentrations (111.1 nM and 333.3 nM), both antibodies demonstrated comparable anti-PD−1 potencies, with EC_50_ values of 11.52 nM for JS207 and 11.62 nM for AK112 (**Figure 5I**).

### 3.15 Thermal stability assessment

The thermal stability is an important factor to be considered during antibody CMC manufacturing and storage. In general, due to multiple domain composition, BsAbs tend to have lower thermal stability compared to conventional monoclonal antibodies that add additional challenge during BsAb development (34, 35). The thermal stability of JS207 was evaluated using two potency assays: anti−PD−1 RGA and anti−VEGFA RGA.

**Figure 6:**
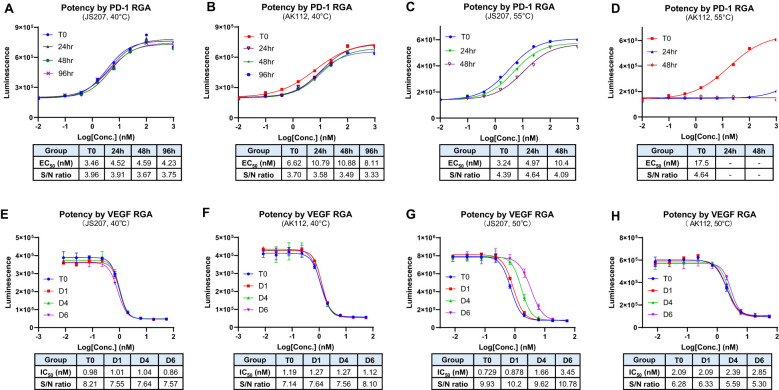
Thermal stability assessment for JS207 using PD-1 RGA and VEGFA RGA. **(A)** Anti-PD-1 potency of JS207 after 40°C stress for 24, 48 and 96 hr. **(B)** Anti-PD-1 potency of AK112 after 40°C stress for 24, 48 and 96 hr. **(C)** Anti-PD-1 potency of JS207 after 55°C stress for 24 and 48 hr. **(D)** Anti-PD-1 potency of AK112 after 55°C stress for 24 and 48 hr. **(E)** Anti-VEGFA potency of JS207 after 40°C stress for 1, 4 and 6 days. **(F)** Anti-VEGFA potency of AK112 after 40°C stress for 1, 4 and 6 days. **(G)** Anti-VEGFA potency of JS207 after 50°C stress for 1, 4 and 6 days. **(H)** Anti-VEGFA potency of AK112 after 50°C stress for 1, 4 and 6 days.

At 40°C, neither JS207 nor AK112 exhibited significant changes in anti−PD−1 potency after 24, 48, or 96 hours of heat stress (**Figures 6A, B**). Similarly, at 40°C, no significant changes in anti−VEGFA potency were observed for JS207 and AK112 after 1, 4, and 6 days of incubation (**Figures 6E, F**). Under extended periods of high temperature (50°C, up to 6 days), JS207 showed a time-dependent increase in IC50 values (potency decrease) but retained an unchanged S/N ratio for anti-VEGFA potency (**Figure 6G**). Under the same stress condition (50°C, up to 6 days), AK112 exhibited increases in IC50 values (potency decrease) and a decrease in the S/N ratio for anti-VEGFA potency (**Figure 6H**).

At 55°C, AK112 lost nearly all anti-PD−1 activity by 24 hours and completely lost anti-PD-1 activity by 48 hours. In contrast, JS207 maintained relatively strong anti−PD−1 activity, retaining 65% potency at 24 hours and 31% at 48 hours compared to time 0. Furthermore, even after 4 hours at 65°C, JS207 retained 15.5% anti-PD-1 activity while AK112 had no activity at all (**Supplementary Figure S2** in **Supplementary Data Sheet**). These results demonstrate that JS207 possesses enhanced thermal stability in anti-PD-1 potency. These results support previous finding that Fab format (anti-PD-1 domain of JS207) is more stable than scFv format (anti-PD-1 domain of AK112) (36).

### 3.16 Anti-tumor efficacy in mouse MC38 tumor model in B-hPD-1 humanized mice

**Figure 7:**
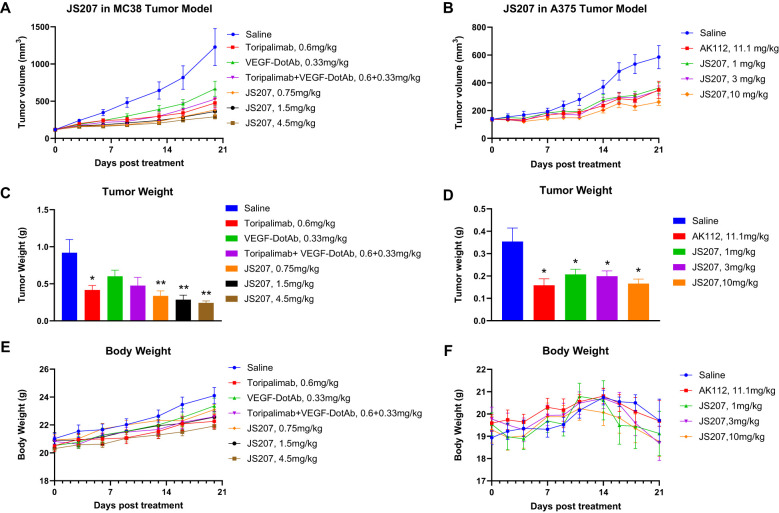
Anti−tumor efficacy of JS207 in the MC38 mouse colon cancer model in C57BL/6-Pdcd1^tm1(PDCD1)Bcgen^/^Bcgen^ mice (B−hPD−1 humanized mice) and the A375 malignant melanoma model in NDG mice. **(A)** Tumor growth curves for MC38 hPD−1 tumors treated with JS207, toripalimab, and toripalimab plus VEGF−DotAb. **(B)** Tumor growth curves for A375 hPD−1 tumors in mice treated with JS207 and AK112. **(C)** Significant decrease in MC38 tumor weight in animals treated with JS207, toripalimab, and toripalimab plus VEGF−DotAb (mean ± SEM, n = 8; *p < 0.05, **p < 0.01 vs Saline). **(D)** Significant reduction in A375 tumor weight in animals treated with JS207 and AK112 (mean ± SEM, n = 8; *p < 0.05 vs Saline). **(E)** Average body weight of animals in the MC38 tumor model. **(F)** Average body weight of animals in the A375 tumor model.

To evaluate the *in vivo* anti−tumor activity of JS207, mouse colon cancer MC38 cells were subcutaneously implanted into C57BL/6-Pdcd1^tm1(PDCD1)Bcgen^/Bcgen humanized mice (abbreviated as B-hPD-1 mice). As shown in **Figures 7A, C**, JS207 significantly inhibited tumor growth in a dose−dependent manner when administered at 0.75, 1.5, and 4.5 mg/kg, achieving tumor growth inhibition (TGI) rates of 76.1%, 78.0%, and 84.4%, respectively, at day 20 post−treatment. At equivalent molar doses, JS207 (0.75 mg/kg) exhibited superior anti−tumor activity compared to toripalimab monotherapy (0.6 mg/kg) or toripalimab plus VEGF−DotAb combination therapy (0.6 mg/kg + 0.33 mg/kg). Notably, none of the treatment groups showed significant body weight loss or other overt side effects, indicating that JS207 was well tolerated (**Figure 7E**).

### 3.17 Anti-tumor efficacy in malignant melanoma A375 model in NDG mice

The anti−tumor efficacy of JS207 was further examined in a human malignant melanoma A375 model using human PBMC transplanted NDG mice. In the saline−treated group, the mean tumor volume reached 585 ± 83 mm³ at day 21. In contrast, mice treated with JS207 at doses of 1, 3, and 10 mg/kg exhibited mean tumor volumes of 362 ± 38 mm³, 344 ± 32 mm³, and 262 ± 25 mm³, corresponding to TGI rates of 49.6%, 53.7%, and 72.0%, respectively (**Figures 7B, D**). Additionally, mice treated with AK112 at 11.1 mg/kg displayed a mean tumor volume of 349 ± 62 mm³ with a TGI of 52.7%. At equivalent molar doses (AK112 at 11.1 mg/kg versus JS207 at 10 mg/kg), JS207 demonstrated superior anti−tumor efficacy compared with AK112. Importantly, the body weights of animals in the treatment groups did not significantly differ from those in the saline group, underscoring the favorable tolerability of both JS207 and AK112 (**Figure 7F**).

## 4 Discussion

The combination of PD-(L)1 and VEGF inhibition has exhibited considerable potential in amplifying antitumor responses. Nevertheless, the co-administration of monoclonal antibodies poses several challenges, including intricate dosing schedules, distinct pharmacokinetics, and increased toxicity risks. Bispecific antibodies (BsAbs) provide a streamlined solution by facilitating dual targeting within a single molecular construct. Interestingly, investigations into HB0025 (anti-PD-L1/VEGFR1 BsAb), CVL006 (anti-PD-L1/VEGFA BsAb) and AK112 (anti-PD-1/VEGFA BsAb) have demonstrated promising preclinical and clinical efficacy, further underscoring the feasibility and potential of this innovative therapeutic approach (31, 33, 37, 38). As summarized in **Table 1**, these BsAbs, whether in an anti-PD-L1/VEGFA format or an anti-PD-1/VEGFA format, can enhance T cell activation and *in vivo* antitumor activity, regardless of the Fc region configuration, whether IgG4, or IgG1 with or without ADCC (antibody-dependent cellular cytotoxicity). This study introduces JS207, a novel BsAb targeting PD-1 and VEGFA, engineered to overcome resistance mechanisms in cancer therapy by concurrently inhibiting immunosuppressive and angiogenic pathways. JS207 demonstrated high-affinity binding to human PD-1 (K_D_ = 4.60×10^-10^ M) and potent inhibition of VEGFA activity (IC_50_ = 0.773 nM), effectively blocking both PD-1/PD-L1 and VEGFA/VEGFR2 interactions. Notably, JS207 exhibited an 11-fold higher PD-1 binding affinity compared to AK112 (K_D_ = 5.05×10^-9^ M) while maintaining comparable VEGFA binding activity. JS207 could bind to human PD-1 either by itself or as JS207/VEGFA complex. Furthermore, JS207 could simultaneously bind to human PD-1 and VEGFA (**Figure 3**). Mechanistically, JS207 induced robust PD-1 internalization, a process augmented by VEGFA, and sustained T-cell activation. In preclinical models, JS207 achieved remarkable anti-tumor efficacy, with tumor growth inhibition (TGI) rates of 84.4% in the MC38 colon cancer model and 72.0% in the A375 melanoma model at 10 mg/kg, outperforming AK112 at equivalent molar doses (11.1 mg/kg). Collectively, these findings underscore JS207’s dual mechanism of action and its promise as a next-generation therapeutic agent in cancer immunotherapy.

**Table 1:** Comparison of JS207, AK112, HB0025 and CVL006.

BsAb	Structural Format	Binding Affinities	Biological Activities
**JS207**	PD-1/VEGFA BsAb IgG4 M.W. = 180 kDa	High affinity for PD-1 and VEGFA KD values by SPR assay ^a^: ▪ PD-1 = 4.60E-10 M ▪ VEGFA = 9.00E-12 M	Enhances T-cell activation, cytokine release, and PD-1 internalization Blocks VEGF-induced HUVEC proliferation Enhances in vivo anti-tumor activities
**AK112**	PD-1/VEGFA BsAb IgG1, ADCC silenced M.W. = 200 kDa	High affinity for PD-1 and VEGFA KD values by SPR assay ^a^: ▪ PD-1 = 5.05E-09 M ▪ VEGFA = <2.59E-11 M	Enhances T-cell activation, PD-1 internalization and inhibits tumor angiogenesis Enhances in vivo anti-tumor activities
**HB0025**	Fusion protein-based PD-L1/VEGFR1 BsAb IgG4 M.W.= 171 kDa	High affinity for PD-L1 and VEGFR1 KD values by SPR assay ^b^: ▪ PD-L1 = 1.76E-9 M ▪ VEGFA = 4.72E-12 M	Enhances T-cell activation Blocks VEGF-induced HUVEC proliferation and migration Enhances in vivo anti-tumor activities
**CVL006**	PD-L1/VEGFA BsAb IgG1, ADCC active M.W. = 150 – 200 kDa ^d^	High affinity for PD-L1 and VEGFA KD values by SPR assay ^c^: ▪ PD-L1 = 1.55E-10 M ▪ VEGFA = 1.50E-11 M	Enhances T-cell activation Blocks VEGF-induced HUVEC proliferation Enhances in vivo anti-tumor activities

^a^ Results of current study; ^b^ Data from Cui et al., 2021 (Reference 36); _c_ Data from Wang et al., 2024 (Reference 37); ^d^ the exact molecular weight is not publicly available.

The TME and aberrant angiogenic signaling represent major hurdles to achieving durable responses with current cancer immunotherapies (39). Although ICIs such as PD-1 blockers (e.g., pembrolizumab) and anti-VEGFA agents (e.g., bevacizumab) have revolutionized cancer treatment, their efficacy is often compromised by resistance mechanisms, including compensatory angiogenic pathways and immune evasion (2, 40). Several studies using patient-derived xenografts and tumor tissue analyses indicate that tumors with elevated VEGFA levels foster a microenvironment rich in PD-1–positive immune cells. For example, Voron et al. demonstrated that VEGFA modulates inhibitory checkpoint expression on CD8^+^ T cells, thereby providing a mechanistic basis for combining anti-angiogenic therapy with immune checkpoint blockade (41). Huang et al. showed that anti-angiogenic treatment normalizes tumor vasculature and reprograms the TME to enhance immune cell infiltration (42). Allen et al. reported that pairing antiangiogenic with anti-PD-L1 therapies synergistically stimulates tumor immunity through complementary mechanisms (43). These findings underscore the translational relevance of our work, suggesting that anti-PD-1/VEGFA BsAbs could potentially generate a more effective antitumor response. Moreover, preclinical and clinical evidence supports the rationale for combining PD-1 and VEGFA blockade, as VEGFA not only drives angiogenesis but also impairs dendritic cell maturation and cytotoxic T-cell activity (25, 41). It has been shown that dual PD-1 and VEGFR-2 blockade promotes vascular normalization and enhances anti-tumor immune responses in murine hepatocellular carcinoma models (23). Clinical studies using combinations such as bevacizumab (anti-VEGFA) with atezolizumab (anti-PD-L1) have validated this approach, although challenges related to discordant pharmacokinetics and overlapping toxicities remain (24).

JS207’s efficacy arises from its ability to engage PD-1 and VEGFA, disrupting two critical TME pathways. Interestingly, our results revealed that exogenous VEGFA enhanced JS207’s cell-based binding (**Figure 4F**) and anti-PD-1 activity (**Figures 4D, E**). Several mechanisms may contribute to this phenomenon: (i) Target Upregulation: VEGFA may upregulate PD-1 expression on target cells, amplifying the impact of dual blockade (21). (ii) Avidity Effects: VEGFA binding could stabilize JS207 near PD-1-expressing cells, increasing local antibody concentration and blockade efficiency (44). (iii) Feedback Modulation: Neutralizing VEGFA may reduce immunosuppressive PD-L1 expression, indirectly enhancing PD-1 blockade (45), (iv) Receptor Clustering: Dual binding may facilitate PD-1 cross-linking and sustaining T-cell activation (37), (vi) Enhanced PD-1 Internalization: JS207 achieved 65% PD-1 internalization in the presence of VEGFA (vs. 32% without) (**Figures 5A–I**), and (vii) Synergistic Pathway Inhibition: Concurrent PD-1/VEGFA blockade elevated IL-2 and IFN-γ secretion in mixed lymphocyte reactions, mirroring effects seen with toripalimab + VEGF-DotAb combination therapy (**Figures 4G, H**).

While BsAbs offer a versatile platform, selecting the optimal BsAb format is critical because its structural design directly impacts efficacy, safety, and CMC manufacturability by influencing production complexity, yield, and scalability. While scFv is widely used in BsAb development (e.g., IgG-scFv) due to its versatility and compact structure, VHH format (e.g., IgG-VHH) could serve as a good alternative because of its superior chemical and physical properties such as smaller size, higher solubility and lower production cost (46). Previous studies have shown that VHH antibodies have better stability compared to scFv antibodies due to the structural differences in domain composition, hydrophobic interactions, disulfide bonds and evolutionary adaptation (36, 47, 48). Our findings demonstrated that anti-PD-1/VEGFA BsAb in an IgG-VHH form like JS207 could simultaneously block PD-1 and VEGFA with high affinity and biological activities while maintaining good thermal stability. In terms of anti-PD-1 activity, JS207 was extremely stable under heat tress retaining good activity after 48 hours at 55°C (**Figure 6C**) and measurable activity after 4 hours at 65°C (**Supplementary Figure S2**). For anti-VEGFA activity, JS207 also showed good heat resistance for extended periods. After 6 days of heat stress, there was no change in its anti-VEGFA potency at 40°C and retained measurable activity at 50°C (**Figures 6E, G**). Thus, JS207 demonstrates an excellent heat stability profile, a critical factor for CMC manufacture, storage and drug shelf life.

The rationale for using NDG mice in the A375 model and B-hPD-1 mice in the MC38 model for JS207 efficacy studies is based on their distinct immunological characteristics. NDG mice are highly immunocompromised, allowing human tumor cell lines such as A375 melanoma to engraft and proliferate without immune rejection. This model helps isolate the effects of the anti-VEGF/PD-1 bispecific antibody on tumor vascularization and growth while eliminating the confounding influence of an active adaptive immune response. In contrast, B-hPD-1 mice carry a human PD-1 gene and support syngeneic MC38 tumors within a fully functional immune system. This setup more accurately mimics the human TME, enabling the evaluation of immune checkpoint inhibition effects on T cell activation, cytokine secretion, and overall antitumor activity. While NDG mice cannot model immune-mediated responses due to their lack of adaptive immunity, B-hPD-1 mice provide a more physiologically relevant immune setting—though many aspects of their biology remain murine. JS207’s preclinical profile positions it as a promising candidate for clinical translation. Delivering dual targeting in a single agent could mitigate toxicity and dosing complexities inherent to combination therapies (28). Key implications include (i) Potency at Lower Doses: JS207 achieved significant TGI at 0.75 mg/kg in the MC38 model, outperforming toripalimab monotherapy (**Figure 7A**); (ii) Broad Applicability: Cross-reactivity with cynomolgus PD-1/VEGFA supports non-human primate toxicology studies; and (iii) Enhanced Stability simplifies storage and distribution requirements (49). These attributes could address unmet needs in oncology, particularly for tumors with high PD-L1/VEGF co-expression or resistance to single-agent immunotherapies (50).

While this study establishes JS207’s therapeutic potential, several limitations warrant attention, such as, (i) Model Constraints: The use of immunocompromised NDG mice limits assessment of adaptive immunity; humanized models with intact immune systems are warranted (51); (ii) Mechanistic Clarity: The structural basis for VEGFA-enhanced PD-1 internalization remains unclear, necessitating crystallography or cryo-EM studies (52). One hypothesis is that VEGFA directly interacts with PD-1 or associated adaptor proteins, triggering conformational changes that promote receptor internalization. Structural studies, such as cryogenic electron microscopy (cryo-EM) and co-crystallization, could elucidate whether VEGFA binds directly to PD-1 or alters the surrounding membrane microenvironment. These methods might reveal specific binding interfaces, conformational shifts, or protein-lipid interactions that facilitate internalization. Future directions include mutagenesis experiments based on structural data to validate these mechanisms, paving the way for targeted therapies that modulate immune checkpoint recycling and optimize the antitumor immune response; and (iii) Biomarker Validation: PD-L1/VEGF co-expression levels should be evaluated as predictive biomarkers in clinical trials (53). Given these encouraging preclinical findings, JS207 is advancing into clinical development to assess safety, pharmacokinetics, and efficacy in cancer patients. Future studies exploring combinations with chemotherapy or immunomodulators (e.g., CTLA-4, BTLA inhibitors) could further enhance therapeutic outcomes (12, 54, 55).

In conclusion, JS207 represents a significant advancement in bispecific antibody therapeutics by integrating potent PD-1 and VEGFA blockade into a single and stable molecule with high affinity for target antigens. Its ability to enhance PD-1 internalization, synergistically inhibit immunosuppressive/angiogenic pathways, and achieve robust anti-tumor efficacy in preclinical models underscores its therapeutic potential. By addressing limitations of existing therapies, such as resistance mechanisms, pharmacokinetic discordance, and formulation instability, JS207 offers a promising strategy for improving outcomes in advanced cancers. Clinical validation is now imperative to translate these preclinical advantages into patient benefits. Given the well-documented toxicity concerns associated with anti-VEGF and anti-PD-(L)1 combination therapy, dual-targeting VEGF/PD-(L)1 BsAb may pose potential immune, vascular, and inflammatory risks. Therefore, careful monitoring, optimized dosing strategies, and rigorous clinical validation are essential to ensuring their safe and effective therapeutic application.

## Data Availability

The original contributions presented in the study are included in the article/**Supplementary Material**. Further inquiries can be directed to the corresponding authors.
