# Supplementary material for: Characterization and functional evaluation of JS207, a novel bispecific antibody against human PD-1 and VEGFA
Source: Front Immunol. 2025 Jun 18;16:1612547. doi: 10.3389/fimmu.2025.1612547 (PMC12213583; doi:10.3389/fimmu.2025.1612547)
Supplement: Supplementary file 1 [file DataSheet1.docx]

**Supplementary Data Sheet**

1. **Supplementary Materials**
   1. **List of materials and reagents used in the study:**

| **Name** | **Manufacturer, Lot #** |
| --- | --- |
| JS207 | Suzhou Junmeng^1^, 22111451 |
| AK112^2^ | Suzhou Junmeng, 20230117 |
| Ak112^3^ | Akesobio, BA112P202405004 |
| Toripalimab^4^ | Suzhou Junmeng, 002-WRS-01 |
| VEGF-DotAb^5^ | Suzhou Junmeng, 20220804 |
| Human PD-1-mFc | Suzhou Junmeng, 20210421 |
| Human PD-1 | Suzhou Junmeng, 20211103 |
| Cynomolgus PD-1 | Sino Biological, LC15SE2304 |
| Rat PD-1 | Sino Biological, LC16DE1904 |
| Mouse PD-1 | R&D Systems, DDJJ0818051 |
| Human/ Cynomolgus VEGFA | Sino Biological, LC14AP0803 |
| Human VEGFA,His tag | Acro, 2410-2069F1-146 |
| Human VEGFB | Acro, 675-223PF1-12C |
| Human VEGFC | Sino Biological, LC16AU0206 |
| Human VEGFD | Sino Biological, LC14JA2103 |
| Human VEGFE | Biorbyt, C9187 |
| Human placental growth factor (PLGF) | Sino Biological, LC16MA1408 |
| Rat VEGFA | Sino Biological, LC12MC2311 |
| Mouse VEGFA | Sino Biological, LC13OC1410 |
| Anti-KLH hIgG4 | Suzhou Junmeng, 20220211 |
| Human PD-L1 | Suzhou Junmeng, 20200309 |
| Human PD-L2 | Sino Biological, LC16MA1426 |
| Human ICOS | Acro, 2702-87TF1-RX |
| Human BTLA | Suzhou Junmeng, 20210713 |
| Human CTLA-4 | Suzhou Junmeng, 20211228 |
| Human CD28 | Suzhou Junmeng, 20200806 |
| Biotin Human PD-L1-hFc | Suzhou Junmeng, 20191011 |
| Human PD-L2-mFc | Novoprotein, 0331547 |
| EasySep Buffer | STEMCELL, 20144 |
| Human CD4 microbeads | Miltenyi Biotec, 130-045-101 |
| BD CBA human IL2 Flex set | BD, 558270 |
| BD CBA human IFNγ kit | BD, 558269 |
| VEGFR2, Human, Biotinylated | Sino Biological, LC13MA1603 |
| Goat anti-human Fc fragment antibody | Jackson ImmunoResearch, 109-005-098 |
| Goat anti-human IgG (Fc specific)-HRP | Sigma, A0170 |
| Mouse Anti-Human IgG4 Fc-HRP | Southern Biotech, 9200-05 |
| Goat anti-mouse IgG (Fc specific) | Sigma, A2554 |
| JS501 (anti-human VEGFA mAb) | Suzhou Junmeng, T202228 |
| Peroxidase Streptavidin | Jackson Immuno, 016-030-084 |
| BSA | Sigma, V900933-1KG |
| TMB | Sigma, T2885 |

^1^Suzhou Junmeng = Suzhou Junmeng Biosciences Co. Ltd.

^2^The material was used as positive control in target antigen binding studies.

^3^The material was used as positive control in potency, PD-1 internalization, and thermal stability studies.

^4^Toripalimab (also known JS001) is a recombinant humanized anti-PD-1 monoclonal antibody developed by Junshi Biosciences (Shanghai, China). The Fab domain of JS207 targeting human PD-1 was derived from toripalimab.

^5^VEGF-DotAb is an anti-human VEGFA heavy chain antibody screened via the DotBody platform (DotBio, Singapore) and produced by Suzhou Junmeng Biosciences. The anti-VEGFA domain of JS207 was derived from VEGF-DotAb.

- 1. **Cell lines:**

For Anti-PD-1 reporter gene assay, Jurkat/PD-1/NFAT-Luc cells and PD-L1 aAPC/CHO-K1 cells were obtained from Promega (USA).

For Anti-VEGFA reporter gene assay, H293/VEGFR2 reporter cells were sourced from the National Institutes for Food and Drug Control (NIFDC, China).

MC38 mouse colon cancer cells were acquired from Cobioer Biosciences Co., Ltd (Nanjing, China).

A375 human melanoma cells were purchased from the American Type Culture Collection (ATCC, USA).

Human peripheral blood mononuclear cells (PBMCs) were provided by Shanghai Aoneng Biosciences (Shanghai, China).

- 1. **Animals:**

NDG mice and C57BL/6-Pdcd1^tm1(PDCD1)Bcgen^/Bcgen (abbreviated as B-hPD-1 mice) were obtained from Biocytogen Inc., (Nantong, China) with animal license No. of SCXK(Su)2016-0004.

1. **Supplementary Methods**
   1. **Enzyme-Linked Immunosorbent Assay (ELISA):**

**Binding to PD-1:** To assess the binding activity of JS207 to PD-1 and related proteins, 0.3 μg/mL of each target protein (PD-1, BTLA, PD-L1, PD-L2 and ICOS, all from human) was coated onto 96‐well plates. After coating, the wells were blocked with 2% bovine serum albumin (BSA). Test samples, a positive control (toripalimab), and a negative control (anti-KLH hIgG4) were then added. Bound antibody was detected using a goat anti‐human IgG (Fc‐specific) peroxidase-conjugated antibody diluted 1:5000. Color development was performed with 0.1 mg/mL tetramethylbenzidine (TMB), and the reaction was terminated with 2 M HCl. Absorbance was measured at 450 nm with a reference wavelength of 620 nm, and the EC_50_ value was determined by fitting the data to a four-parameter logistic (4PL) model. To assess the species cross-reactivity of JS207, PD-1 proteins from different species, rat, mouse, and Cynomolgus monkey were used.

**Binding to VEGFA:** To evaluate the binding of JS207 to VEGFA, 0.3 μg/mL of VEGFA protein (from human, monkey, rat, or mouse) was coated onto 96‐well plates and blocked with 2% BSA. Serial dilutions of the test samples and the negative control (anti-KLH hIgG4) were then added. Detection was carried out using the same goat anti‐human IgG (Fc‐specific) peroxidase antibody (diluted 1:5000), followed by color development with 0.1 mg/mL TMB and termination of the reaction with 2 M HCl. Absorbance was read at 450 nm/620 nm. EC_50_ values were derived using the 4PL model using GraphPad Prism 10. The signal to noise (S/N) ratio was generated by dividing the Top response by Bottom response.

**Blocking the interaction between human PD-1 with PD-L1/PD-L2:** Competitive inhibition of the interaction between human PD-1 and its ligands (PD-L1 and PD-L2) was assessed using blocking ELISAs. Human PD-1 protein (1.5 μg/mL) was coated on the plate and blocked with 2% BSA. Serial dilutions (starting at 100 μg/mL, with a 3‐fold dilution series) of JS207, toripalimab, AK112, and anti-KLH hIgG4 were prepared in assay buffer containing biotin‐PD-L1 hFc (4.0 μg/mL) and added to the wells to allow competitive binding. Detection was performed using peroxidase‐conjugated streptavidin diluted 1:5000. Following color development with 0.1 mg/mL TMB and reaction termination with 2 M HCl, the absorbance was measured at 450 nm/620 nm.

**Blocking the interaction between human VEGFA and VEGFR2:** For the assessment of JS207’s competitive inhibition of the VEGFA–VEGFR2 interaction, 0.5 μg/mL of human VEGFA was coated on 96‐well plates and blocked with 2% BSA. Serial dilutions (starting at 400 μg/mL, 3‐fold dilution series) of JS207, VEGF-DotAb, AK112, and anti-KLH hIgG4 were added in assay buffer containing biotin‐VEGFR2 (0.3 μg/mL). After incubation, peroxidase‐conjugated streptavidin (diluted 1:5000) was used for detection. Color development was conducted with 0.1 mg/mL TMB, the reaction halted with 2 M HCl, and absorbance was measured at 450 nm/620 nm.

- 1. **Surface Plasma Resonance (SPR) Binding Assays**

**Binding to human PD-1:** The binding affinity of JS207 to human PD-1 protein was measured by Biacore T200. 40 µg/mL of anti-human Fc fragment antibody was coated to CM5 chip, and then 2 μg/mL of JS207, AK112 and toripalimab were captured, respectively. Serially diluted human PD-1 at 140 nM, 70 nM, 35 nM, 17.5 nM, 8.75 nM and 4.375 nM was then applied. The kinetic model was analyzed using the Biacore analysis software (Biacore T200 Evaluation Software 3.0), and the binding affinity (K_D_ value) was calculated.

**Binding to human VEGFA:** The binding affinity of JS207 to human VEGFA protein was determined by the Biacore T200. 40 µg/mL of anti-human Fc fragment antibody was coated to CM5 chip, and then 2 µg/mL of JS207, AK112 and 0.4 µg/mL of VEGF-DotAb (anti-human VEGFA DotAb) were added. When testing JS207 and VEGF-DotAb, human VEGFA protein was diluted to 6 nM, 3 nM, 1.5 nM, 0.75 nM, 0.375 nM and 0.188 nM; when testing AK112, human VEGFA protein was diluted to 12 nM, 6 nM, 3 nM, 1.5 nM, 0.75 nM and 0.375 nM. The kinetic model was analyzed using Biacore analysis software Biacore T200 Evaluation Software 3.0, and the binding affinity (K_D_ value) was calculated.

**Binding of JS207/VEGFA complex to human PD-1:** The binding of the pre-formed JS207/VEGFA complex to human PD-1 was assessed using an Octet RED96e system (Sartorius Fortebio). In this assay, 20 µg/mL of human PD-1-mFc was immobilized on an AMC biosensor. The sensor was then incubated with JS207, AK112, the JS207/VEGFA complex, or the AK112/VEGFA complex. Complexes were generated by pre-incubating JS207 or AK112 with VEGFA at a 1:2 molar ratio at room temperature for 30 minutes prior to analysis. Kinetic parameters were analyzed using Data Analysis 11.1 software (Fortebio), and the K_D_ values were derived accordingly.

**Simultaneous binding of JS207 to human PD-1 and VEGFA:** The ability of JS207 to simultaneously bind to human PD-1 and human VEGFA was measured using the Biacore T200. Two different experimental formats were used: (1) PD-1 First, then VEGFA: 20 µg/mL anti-mouse Fc antibody was coupled to the CM5 chip, and 2 μg/mL human PD-1 with a mouse Fc was captured. 100 nM of JS207, AK112, toripalimab and buffer were injected, respectively. Followed by 80 nM of unlabeled VEGFA or buffer. (2) VEGFA First, then PD-1: 20 µg/mL anti-his tag antibody was coupled to the CM5 chip, and 0.1 µg/mL human VEGFA with His tag was captured. 500 nM of JS207, AK112, VEGF-DotAb and buffer were injected, respectively. Followed by 300 nM of human PD-1 with mouse Fc or buffer.

- 1. **Luciferase Reporter Gene Assays**

**PD-1 reporter gene assay:** Two engineered cell lines were used in this assay: Jurkat/PD-1-NAFT-Luc effector cell in which human PD-1 and luciferase reporter gene coupled with NFAT were stably expressed, and PD-L1 aAPC/CHO-K1 target cell in which human PD-L1 was stably expressed. JS207 can inhibit the binding of PD-1 in Jurkat/PD-1 cells to PD-L1 in CHO/PD-L1 cells. The biological activity (potency) of the anti-PD-1 arm of JS207 was measured by detecting the NFAT/luciferase reporter gene activation via bioluminescent measurement. EC50 values were derived using the 4PL model with GraphPad Prism 10. The signal to noise (S/N) ratio was generated by dividing the Top response by Bottom response.

**VEGF reporter gene assay:** H293/VEGFR2 cell that was engineered to express VEGFR2/NFAT-luciferase was used in this assay. When VEGFA binds to VEGFR2 to initiate the signaling pathway, the transcription factor NFAT is activated to express the luciferase reporter gene, and chemiluminescence is generated after the addition of substrate. JS207 inhibits the VEGFA binding to VEGFR2 reducing the luciferase reporter expression. Thus, the biological activity (potency) of the anti-VEGFA domain of JS207 can be measured by detecting the bioluminescent signal. IC50 values were derived using the 4PL model with GraphPad Prism 10. The signal to noise (S/N) ratio was generated by dividing the Top response by Bottom response.

- 1. **HUVEC proliferation inhibition assay**

Human umbilical vein endothelial cells (HUVECs) and recombinant human VEGFA protein were used to evaluate the effect of JS207 on VEGF-mediated HUVEC proliferation. VEGFA at 10 ng/mL was added to a 96-well plate, and the test antibody solutions (JS207, AK112, VEGF-DotAb and anti-KHL-IgG4) at 0.004 nM-10 nM were added to the plate and incubated at 37 °C for 30 minutes. Then, HUVECs at 3×10^4^ cells/mL, 100 µL/well were seeded into the assay plate and incubated at 37 °C for 96 hours. After incubation, cell counting-Lite luciferase assay reagent was added and chemiluminescence signals were measured using a multifunctional microplate reader. IC50 values were derived using the 4PL model with GraphPad Prism 10. The signal to noise (S/N) ratio was generated by dividing the Top response by Bottom response.

- 1. **Flow Cytometry Experiments**

**Internalization assay using cell surface residual PD-1 quantification method:** H293/PD-1 cells were prepared at a concentration of 2×10⁶ cells/mL, and 50 µL per well was seeded in a 96-well plate. JS207, AK112, and toripalimab were serially diluted (starting at 80 nM with 3-fold dilutions) and added to the wells in the absence or presence of VEGFA (2X; starting at 160 nM with 3-fold dilutions). After a 30-minute incubation at 4 °C, the cells were divided into two aliquots and incubated at 37 °C and 4 °C for 0.5, 1, 2, and 4 hours, respectively. Subsequently, all cells were stained with an anti-human IgG-PE antibody for 30 minutes at 4 °C. The samples were then analyzed by flow cytometry. The mean fluorescence intensity (MFI) of the PE channel was determined, and the internalization index was calculated using the following formula**:**

Internalization Index = [1 - (MFI at 37°C) / (MFI at 4°C)] x 100.

**Internalization assay using intracellular fluorescence method:** JS207, AK112, JS501 and toripalimab were conjugated with CypHer5E using the CypHer5E Mono NHS Ester kit follow manufacture’s instruction (GE Healthecare PA15401). The conjugated antibodies were serially diluted in RPMI 1640 supplemented with 10% FBS to yield final concentrations ranging from 0 to 70 nM (5-fold dilution series), in the absence or presence of VEGFA (2X; final concentration 140 nM). The diluted conjugates were then incubated with PD-1–expressing Jurkat cells (seeded at 1×10⁵ cells per well) for 4 hours at either 4 °C or 37 °C. After incubation, cells were washed with cold medium to minimize further cellular activity and subsequently stained with a commercially available PE-labeled noncompetitive anti-PD-1 antibody (MIH4 PD-1 PE, BD Biosciences 557946). Samples were then analyzed on a BD FACSCanto II flow cytometer (BD Bioscience). Data analysis was performed by FlowJo 10.10.0 (TreeStar) and GraphPad Prism 10.

**Cell-based PD-1 binding:** To assess the effect of VEGFA on cell-based PD-1 binding by JS207, PD-1-expressing Jurkat cells were seeded at 1×10⁵ cells per well in a 96-well plate. Serial dilutions of JS207, AK112, toripalimab, and the negative control anti-KLH hIgG4 (starting at 222.3 nM with two-fold dilutions) were added in the absence or presence of serially diluted VEGFA (starting at 444.6 nM with two-fold dilutions). The cells were incubated at 4 °C for 30 minutes. Following a washing step, the cells were stained with 0.5% (v/v) mouse anti-human Fc-PE antibody for 30 minutes at 4 °C and then analyzed by flow cytometry. Data analysis was performed using FlowJo and GraphPad Prism.

- 1. **Mixed Lymphocyte Reaction (MLR) Assay**

The effect of JS207 on T cell activation measured by the release of IL-2 and IFN-γ was evaluated using a mixed lymphocyte reaction (MLR) system. Cryopreserved peripheral blood mononuclear cells (PBMCs) from different donors were thawed and resuspended in EasySep buffer. CD4⁺ T cells were isolated using the human CD4 microbeads kit (Miltenyi Biotec). Mature dendritic cells (mDCs) and purified CD4⁺ T cells were then seeded into 96-well plates at densities of 10,000 mDCs/well and 100,000 CD4⁺ T cells/well, respectively. Test agents including JS207, AK112, toripalimab, VEGF-DotAb, a combination of toripalimab + VEGF-DotAb, and anti-KLH hIgG4 (negative control) were added to the wells. Each reagent was applied as a serial dilution series with final concentrations ranging from 150 nM to 15 pM. The cells were incubated at 37 °C for 5 days. Supernatants were collected on days 3 and 5, and levels of IL-2 and IFN-γ were quantified using the BD CBA Human IL-2 and IFN-γ kits, respectively.

- 1. **In Vivo Anti-tumor Activity Studies**

**Mouse colon cancer MC38 model in B-hPD-1 humanized mice:** Mouse colon cancer MC38 cells were resuspended in DMEM basal medium and implanted subcutaneously in the right flank of B-hPD-1 humanized mice. Each mouse received 1×10⁶ cells in a 0.1 mL volume. When the mean tumor volume reached approximately 115 mm³, mice were randomly assigned into seven groups (n = 8 per group): (1) saline, (2) toripalimab 0.6 mg/kg, (3) VEGF-DotAb 0.33 mg/kg, (4) toripalimab 0.6 mg/kg + VEGF-DotAb + 0.33 mg/kg, (5) JS207 0.75 mg/kg, (6) JS207 1.5 mg/kg, and (7) JS207 4.5 mg/kg. Treatments were administered intraperitoneally twice weekly for a total of 6 doses. Tumor volumes and animal body weights were recorded twice weekly throughout the dosing period and prior to euthanasia.

**Malignant melanoma A375 model in NDG mice:** Human malignant melanoma A375 cells were suspended in DMEM and mixed with Matrigel at a 1:1 ratio. A total of 5×10⁶ cells in 0.2 mL were implanted subcutaneously into the right flank of NDG mice. When the average tumor volume reached approximately 137 mm³, 10×10⁶ PBMCs in 0.2 mL were injected intravenously. Two days after PBMC administration, the tumor-bearing mice were randomly assigned to five groups (n = 8 per group): (1) saline control group, (2) AK112 11.1 mg/kg, (3) JS207 1.0 mg/kg), (4) JS207 3.0 mg/kg, and (5) JS207 10.0 mg/kg. All the treatments were administered intraperitoneally twice a week for a total of 6 doses. Tumor volumes were measured three times per week, and animal body weights were recorded prior to the first dose, twice weekly during treatment, and immediately prior to euthanasia. Tumor growth inhibition rate (TGI_TV_) was calculated as:

TGI_TV_ (%) = [1-(Ti-T0)/(Vi-V0)]×100%,

Where:

Ti = mean tumor size of the treatment group on the i-th day of administration,

T0 = mean tumor size of the treatment group on day 0 of administration,

Vi = mean tumor size of the negative control group on the i-th day of administration,

V0 = mean tumor size of the negative control group on day 0 of administration.

1. **Supplementary Figures**
   1. **The impact of VEGFA on cell-based PD-1 binding:**

**Figure S1.** VEGFA enhances JS207 PD-1 binding in Jurkat/PD-1 cells. Jurkat/PD-1 cells were seeded at 1×10⁵ cells/well in a 96-well plate. Test samples (JS207, AK112, toripalimab, JS207 + VEGFA, AK112 + VEGFA, toripalimab + VEGFA) were added and the cells were incubated at 4 °C for 30 minutes, stained with mouse anti-human Fc-PE antibody for 30 minutes at 4 °C and then analyzed by flow cytometry. VEGFA enhanced both JS207 and AK112’s PD-1 binding on Jurkat/PD-1 cells.

- 1. **Thermal stability assessment for JS207:**


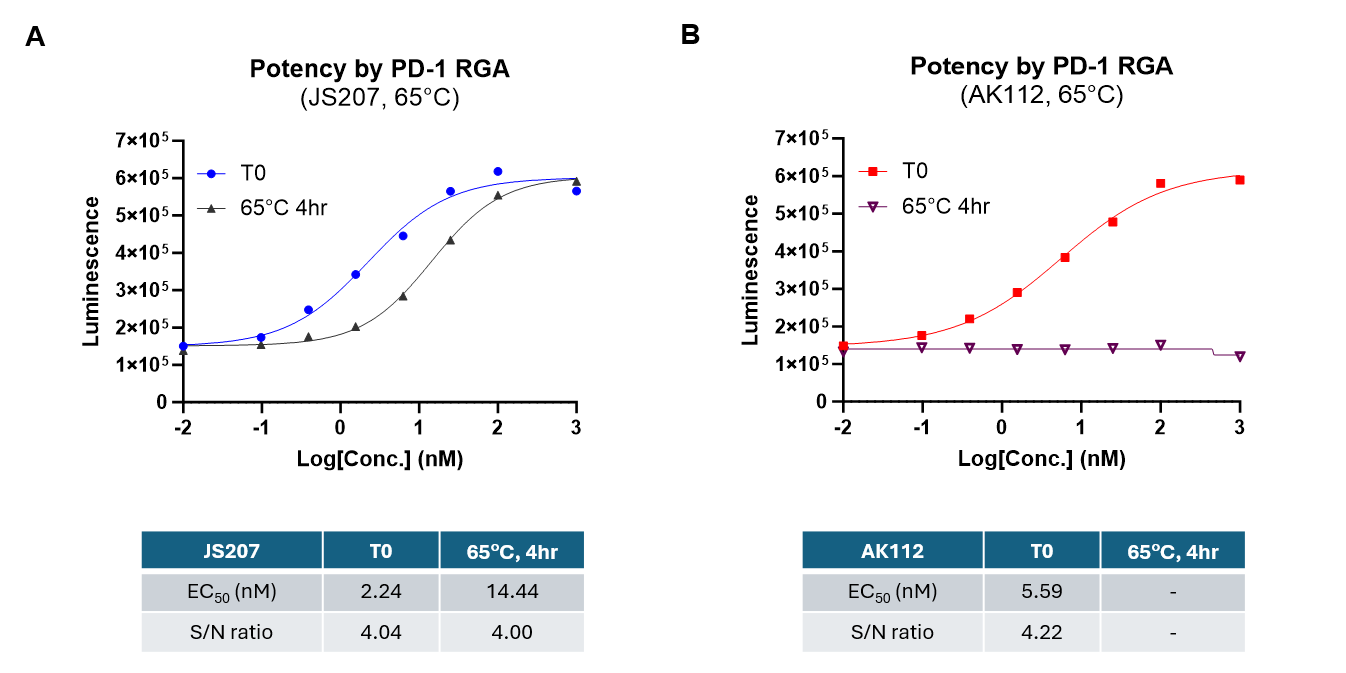


**Figure S2.** Thermal stability of JS207. Test samples were first diluted to 2 mg/mL in cell culture medium and then subjected to heat stress at 65 °C for 0 – 4hr. (A) JS207 showed decreased but measurable potency (15.5% potency) compared to time 0. (B) There was no potency detected for AK112 after 4 hr heat stress.
